# Reduction in Hospital System Opioid Prescribing for Acute Pain Through Default Prescription Preference Settings: Pre–Post Study

**DOI:** 10.2196/24360

**Published:** 2021-04-14

**Authors:** Benjamin Heritier Slovis, Jeffrey M Riggio, Melanie Girondo, Cara Martino, Bracken Babula, Lindsey M Roke, John C Kairys

**Affiliations:** 1 Office of Clinical Informatics Thomas Jefferson University Philadelphia, PA United States; 2 Department of Emergency Medicine Thomas Jefferson University Philadelphia, PA United States; 3 Department of Medicine Thomas Jefferson University Philadelphia, PA United States; 4 Information Services and Technology Thomas Jefferson University Philadelphia, PA United States; 5 Department of Surgery Thomas Jefferson University Philadelphia, PA United States

**Keywords:** informatics, electronic health record, opioids, prescriptions, oxycodone

## Abstract

**Background:**

The United States is in an opioid epidemic. Passive decision support in the electronic health record (EHR) through opioid prescription presets may aid in curbing opioid dependence.

**Objective:**

The objective of this study is to determine whether modification of opioid prescribing presets in the EHR could change prescribing patterns for an entire hospital system.

**Methods:**

We performed a quasi-experimental retrospective pre–post analysis of a 24-month period before and after modifications to our EHR’s opioid prescription presets to match Centers for Disease Control and Prevention guidelines. We included all opioid prescriptions prescribed at our institution for nonchronic pain. Our modifications to the EHR include (1) making duration of treatment for an opioid prescription mandatory, (2) adding a quick button for 3 days’ duration while removing others, and (3) setting the default quantity of all oral opioid formulations to 10 tablets. We examined the quantity in tablets, duration in days, and proportion of prescriptions greater than 90 morphine milligram equivalents/day for our hospital system, and compared these values before and after our intervention for effect.

**Results:**

There were 78,246 prescriptions included in our study written on 30,975 unique patients. There was a significant reduction for all opioid prescriptions pre versus post in (1) the overall median quantity of tablets dispensed (54 [IQR 40-120] vs 42 [IQR 18-90]; *P*<.001), (2) median duration of treatment (10.5 days [IQR 5.0-30] vs 7.5 days [IQR 3.0-30]; *P*<.001), and (3) proportion of prescriptions greater than 90 morphine milligram equivalents/day (27.46% [10,704/38,976; 95% CI 27.02%-27.91%] vs 22.86% [8979/39,270; 95% CI 22.45%-23.28%]; *P*<.001).

**Conclusions:**

Modifications of opioid prescribing presets in the EHR can improve prescribing practice patterns. Reducing duration and quantity of opioid prescriptions could reduce the risk of dependence and overdose.

## Introduction

The opioid epidemic is a public health emergency unlike any other [1-3]. The roots of this crisis are founded in over 30 years of influential guidelines, marketing campaigns, and advertising that led to an increase in opioid prescriptions [4-8]. Overprescribing has increased since the early 1990s [9]. In 2016, there were 66.5 opioid prescriptions written for every 100 persons in the United States [10]. Prescription opioid abuse has been associated with progression to use of heroin [11], and the increased availability of both prescription and illicit opioids has led to a rise in the rate of death [12]. Opioids caused over 67% of drug-related deaths in 2017 and are responsible for nearly 400,000 deaths overall since 1999 [12].

Reductions in prescribing rates have become paramount to combating the opioid epidemic. Higher doses and longer durations of opioid therapy have been associated with an increased risk of chronic opioid use, with a significant increase in risk on treatment days 5 and 31 [13-15]. Therefore, the Centers for Disease Control and Prevention (CDC) recommends that caution be exercised when increasing doses to greater than 50 morphine milligram equivalents (MME) per day, and doses greater than 90 MME/day should be avoided. The CDC also states that 3 days or less is often sufficient for acute pain, and more than 7 days is “rarely needed” [16,17].

The HITECH act of 2009 made electronic health records (EHRs) ubiquitous. By 2017, 95% of hospitals in the United States are using EHRs [18]. EHRs and the technologies associated with them have been successfully utilized to combat the opioid epidemic. Electronic prescribing of controlled substances can improve medication safety, with a 2017 study demonstrating that an increasing number of prescribers are prescribing electronically [19]. Prescription drug monitoring programs, which require prescribers to review prior controlled substance prescriptions prior to prescribing, have shown reductions in opioid prescribing rates [20]. Computerized provider order entry (CPOE) systems are components of modern EHRs, and allow for reduction in practice variation and medical error by simplifying the prescribing of medications to an electronic process [21].

In order to curb overprescribing and reduce quantities of tablets, CPOE-linked interventions focus on preset defaulted prescription settings [22-24]. Previous studies have shown that the introduction or modification of opioid prescription presets has demonstrated reductions of tablet quantities and prescription MME for postsurgical patients [22], as well as significant reductions in the number of tablet quantities dispensed in the emergency department (ED) [23,25]. These studies suggest that modification of EHR-linked CPOE settings is a simple, inexpensive, and effective method of reducing the number of tablets associated with opioid prescriptions. However, all previous studies have focused on isolated emergency [23-27] or surgery departments [22]. We are unaware of any research examining the effects of modifications to opioid presets across an entire hospital system.

Slovis et al [27] previously demonstrated a reduction in duration of therapy and number of tablets dispensed when modifying the opioid prescription presets in our ED [27]. The success of this project prompted an enterprise-wide intervention to modify opioid prescription settings in our EHR’s CPOE.

Our institution’s CPOE consists of 4 fields for oral tablet prescription entry: (1) dose (number of tablets or milligrams per dose), (2) frequency (doses per day), (3) duration (number of days), and (4) quantity (number of tablets per prescription). Prior to our intervention dose, frequency and quantity were required for prescribing, but duration was not.

In August 2018, we implemented a number of interventions in our EHR’s CPOE: (1) we made the duration of treatment for an opioid prescription mandatory, (2) we provided a quick button for 3 days’ duration on all opioid prescriptions while removing all other quick buttons, and (3) we set the default quantity to 10 tablets for all oral opioid formulations, and removed any departmental variations. Prior to our intervention there was variability in durations and quantity dispensed for opioids (Multimedia Appendix 1). All interventions were passive in nature, no decision support alerts were part of the design, and there were no provider re-education measures nor large-scale announcement as part of the implementation design.

The purpose of this study was to assess the effects of modifications of opioid prescription default settings across an entire institution. We hypothesize that modifications of these settings can lead to reductions in duration of treatment, tablets dispensed, and proportion of prescriptions greater than 90 MME/day for a hospital system for patients with nonchronic pain. By demonstrating a reduction in prescribing at the hospital system level we have the potential to reduce risk of dependence, overdose, and possibly death.

## Methods

### Design, Setting, and Participants

In this quasi-experimental retrospective pre–post analysis, we examined the effects on prescribing patterns at our institution before and after modifications of opioid prescription settings. Thomas Jefferson University Hospitals, as an enterprise, has 908 acute care beds and over 2700 physicians and practitioners caring for more than 1.4 million people throughout the inpatient, outpatient, and ED settings [28]. This study was performed at the Center City Division, our urban academic institution comprising a large tertiary-care hospital, a community hospital, and multiple ambulatory clinics. Our study encompasses all outpatient prescriptions for opioids written between September 1, 2017, and August 31, 2019, in our EHR (Epic Systems Corporation) and the patients who received them. We did not include prescriptions for buprenorphine or methadone as these medications are used in medical assisted treatment (MAT) for opioid use disorder [29]. We limited our analysis to only oral capsule and tablet formulations (excluding oral liquid, buccal films, patches, etc.).

Our interest was in the effect of our intervention on opioids prescribed for acute pain. To this end, we excluded patients with chronic pain from our analysis via a modified approach to a validated algorithm [30]. Tian et al [30] achieved an accuracy of 95% for the identification of patients with chronic diseases via (1) a single International Classification of Diseases, 9th Revision (ICD-9) code [31] “highly likely” to represent chronic pain OR (2) 2 or more ICD-9 codes “likely” to represent chronic pain separated by at least 30 days OR (3) receipt of at least 90 days of opioids OR (4) an ICD-9 code “likely” to represent chronic pain AND 2 or more pain scores greater than or equal to 4.

We mapped ICD-9 codes utilized in the algorithm to the 10th Revision (ICD-10) [32] using the Centers for Medicare and Medicaid Services General Equivalence Mappings [33] via the R package “touch” [34]. ICD code mappings were also manually reviewed. There were 9476 prescriptions in our cohort that did not include an ICD-10 and remained in our analysis as if they did not meet criteria for exclusion. Our data set included unreliable pain score documentation, so we eliminated this step in the algorithm. Pain scores held the lowest positive predictive value for identifying patients with chronic pain in the initial study [30], and we presumed that excluding these patients would only bias our results against our objective, as patients with chronic pain are presumed to have long-term prescriptions. Finally, we excluded all prescriptions written in the ED given the results of our previous intervention.

### Variables

Using our analytics software Qlik Sense (QlikTech International), we identified all prescriptions for non-MAT opioid medications written during the study period and extracted a number of variables for each individual prescription and the associated patient.

At the prescription level these variables were medication, number of tablets, dosage unit (ie, mg), route of administration (oral vs rectal), and frequency of administration. Using our previously described method, we also included the calculated duration of therapy [35]. We utilized this value to represent the duration of the prescription as our prior work demonstrated that what is documented in the prescription is at times unreliable [35]. We also extracted precalculated MME/day for each prescription. At the patient level we extracted demographic data including age and sex.

### Data Cleaning, Outcomes, and Statistical Analysis

In order to mitigate bias, all information was obtained through the same data query, and all participants were selected in the same way (eg, a script for an opioid medication). Participants were retrospectively recruited from a continuous 24 months.

Participants were divided into 2 cohorts (12 months before and after the intervention). The intervention was introduced on August 24, 2018, so the month of August 2018 was included in the preintervention cohort to avoid influencing postintervention results.

We calculated and compared frequencies of missing values for metrics of interest before and after the intervention. Demographic information was compared to assess similarity between the 2 cohorts.

The total number of prescriptions and unique patients were evaluated before and after the intervention. To ensure our intervention did not inadvertently cause an increase in the frequency of opioid prescribing, we compared the median number of prescriptions per patient per month as well as the odds of a patient receiving more than 1 prescription or refills.

The proportion of prescriptions was calculated for each class of opioid medication. Opioid class was determined by the active ingredient in the compound. We chose not to separate by schedule class as these can change [36,37]. We compared the proportions of opioid classes before and after the intervention and then measured if our intervention changed the quantity, duration, or proportion of prescriptions greater than 90 MME/day for all prescriptions, as well as for each opioid class.

For statistical analysis, Wilcoxon rank-sum test was utilized for nonparametric data, and *t* test for parametric data. Chi-square and Fisher exact test were performed for comparison of categorical values. Confidence intervals were included for all appropriate analysis. Statistics were performed in R statistical software (R Core Team).

## Results

### Overview of Prescriptions

There were 128,933 non-MAT opioid prescriptions written during our study period. Of these, 103,338/128,933 (80.15%) were written for oral tablet or capsule formulations. We excluded 23,961/103,338 (23.19%) prescriptions that were written for patients with chronic pain. Of the remaining 79,377, we excluded 1131/79,377 prescriptions (1.42%) that were written by the ED. The median duration for these ED prescriptions was 2.7 days (IQR 2.5-3.3) with a median dispensed quantity of 9 tablets (IQR 9-31).

**Figure 1:**
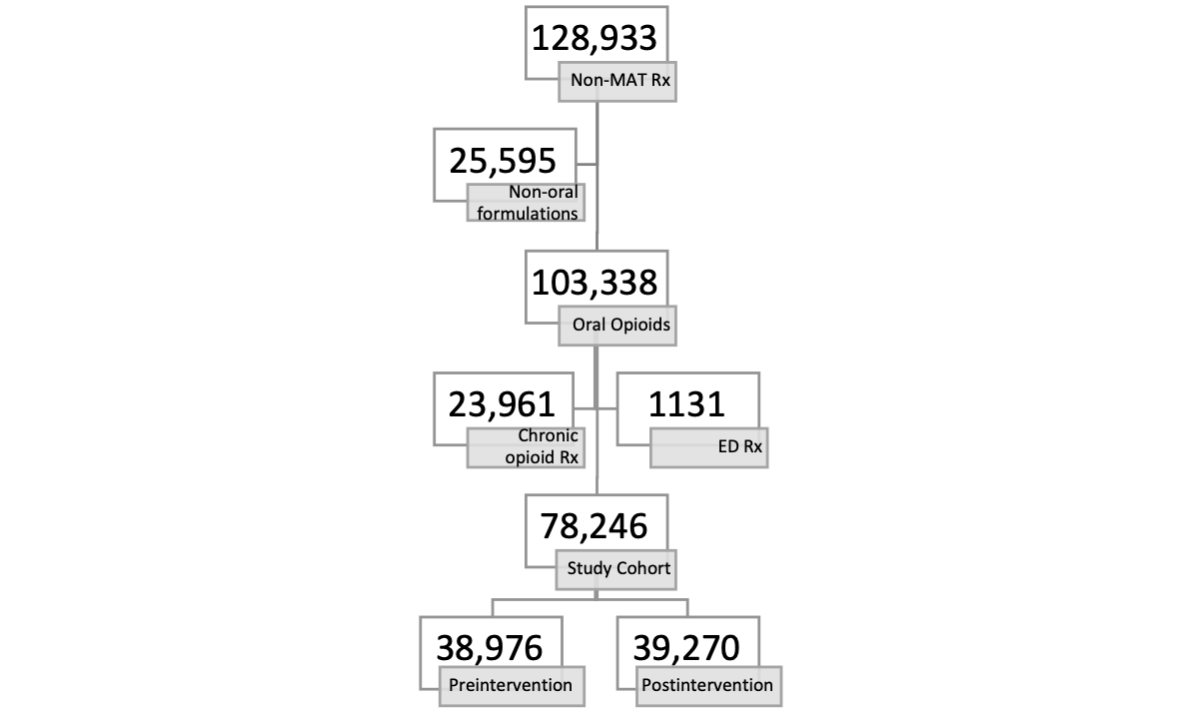
Breakdown of our study cohort included excluded groups. ED: emergency department; MAT: medical assisted treatment.

The remaining 78,246 prescriptions were for 30,975 unique patients. There were 38,976/78,246 (49.81%) prescriptions written for 16,464/30,975 (53.15%) patients in the preintervention period and 39,270/78,246 (50.19%) prescriptions written for 17,399/30,975 (56.17%) patients in the postintervention period. Figure 1 demonstrates the inclusion and exclusion criteria leading to our study cohorts.

### Missing Data

Of the 78,246 prescriptions, 7167 (9.16%; 95% CI 8.96-9.36) were missing a calculated duration. The proportion of missing calculated durations decreased from 9.91% (7758/78,246; (95% CI 9.62-1.02) to 8.41% (6578/78,246; 95% CI 8.14-8.70; *P*<.001) before and after the intervention. In addition, 7156/78,246 (9.15%; 95% CI 8.94-9.35) prescriptions were missing documented quantity dispensed with a reduction from 9.90% (7750/78,246; 95% CI 9.60-10.20) to 8.40% (6570/78,246; 95% CI 8.13-8.68; *P*<.001) before versus after the intervention. Finally, 1139/78,246 (1.46%; 95% CI 1.37-1.54) prescriptions were missing the MME/day field with a significant increase in this proportion before versus after the intervention (915/78,246 [1.17%; 95% CI 1.07-1.28] to 1358/78,246 [1.74%; 95% CI 1.61-1.87]; *P*<.001). Missing values were excluded from their respective analysis.

### Patient Demographics

Overall, 17,344/30,975 (55.99%; 95% CI 55.44%-56.55%) of all unique patients were female. There was no significant difference in patient sex before and after the intervention (17,300/30,975 [55.85%; 95% CI 55.10%-56.61%] vs 17,237/30,975 [56.65%; 95% CI 55.91%-57.39%] female; *P*=.14). The median age was 59, which did not change.

### Rate of Prescribing

There was no significant change in the median number of prescriptions written per month, before and after the intervention (3254.5 [IQR 3176.25-3331.25] vs 3338 [3157.0-3391.5]; *P*=.59). The median monthly prescriptions per person remained 1 (IQR 1-2). The odds of getting more than 1 opioid prescription after the intervention did not significantly increase (1.003, 95% CI 0.967-1.04) and the odds of being prescribed a refill decreased (0.801, 95% CI 0.747-0.858).

### Types of Opioids Prescribed

The majority (48,261/78,246, 61.68%; 95% CI 61.34%-62.02%) of prescriptions during the study period were for oxycodone. Table 1 demonstrates the proportion of individual opioids.

There were small but significant reductions in the proportion of prescriptions for morphine—6.30% (2456/38,976; 95% CI 6.06%-6.55%) to 5.95% (2335/39,270; 95% CI 5.72%-6.19%); *P*=.04)—and oxymorphone—0.37% (143/38,976; 95% CI 0.31%-0.43%) to 0.24% (96/39,270; 95% CI 0.20%-0.30%; *P*=.002). There was no change in the proportion of the other opioids.

**Table 1:** Proportion of each opioid during the study period with 95% confidence intervals.

Medication	Proportion^a^ (N=78,246), n (%)	95% CI
Codeine	379 (0.48)	0.44-0.54
Hydrocodone	13 (0.02)	0.009-0.03
Hydromorphone	3995 (5.11)	4.95-5.26
Meperidine	40 (0.05)	0.04-0.07
Morphine	4791 (6.12)	5.96-6.29
Oxycodone	48,261 (61.68)	61.34-62.02
Oxymorphone	239 (0.31)	0.27-0.35
Tapentadol	172 (0.22)	0.19-0.26
Tramadol	20,356 (26.02)	25.71-26.32

^a^Proportion of the number of that individual opioid prescriptions from the total 78,246 opioid prescriptions.

### Dispensing and Duration

**Figure 2:**
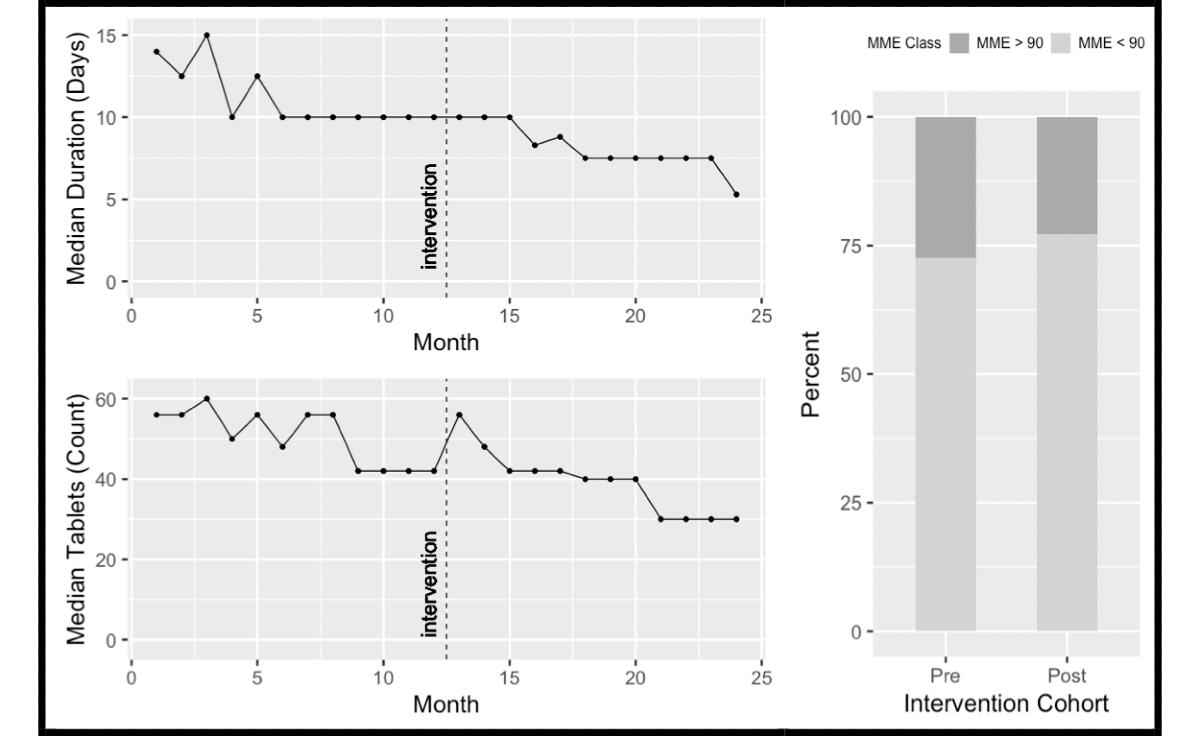
Monthly median tablets prescribed pre and post-intervention, monthly median duration (days) pre and post-intervention and proportion of prescriptions >90 MME/day pre and post-intervention for all opioids. MME: morphine milligram equivalents.

There was a significant reduction in the overall median quantity of opioid tablets dispensed before versus after the intervention (54 [IQR 40-120] vs 42 [IQR 18-90]; *P*<.001). There was also a reduction in median duration of treatment before and after the intervention (10.5 days [IQR 5.0-30] vs 7.5 days [IQR 3.0-30]; *P*<.001). Finally, there was a significant reduction in the proportion of prescriptions greater than 90 MME/day (27.46% [10,704/38,976; 95% CI 27.02%-27.91%] vs 22.86% [8979/39,270; 95% CI 22.45%-23.28%]; *P*<.001), despite no change in the median of 45 MME/day per prescription before and after the intervention. These results are displayed in Figure 2.

There was a significant reduction in all metrics for oxycodone. Tramadol and codeine demonstrated a reduction in tablets dispensed, while codeine also had a reduction in the duration of treatment (*P*<.001 for all three metrics). Hydromorphone had a significant reduction (*P*<.001) in MME greater than 90/day despite no change in the other metrics. Table 2 demonstrates the effect of our intervention overall, and on each type of opioid medication before and after the intervention.

**Figure 3:**
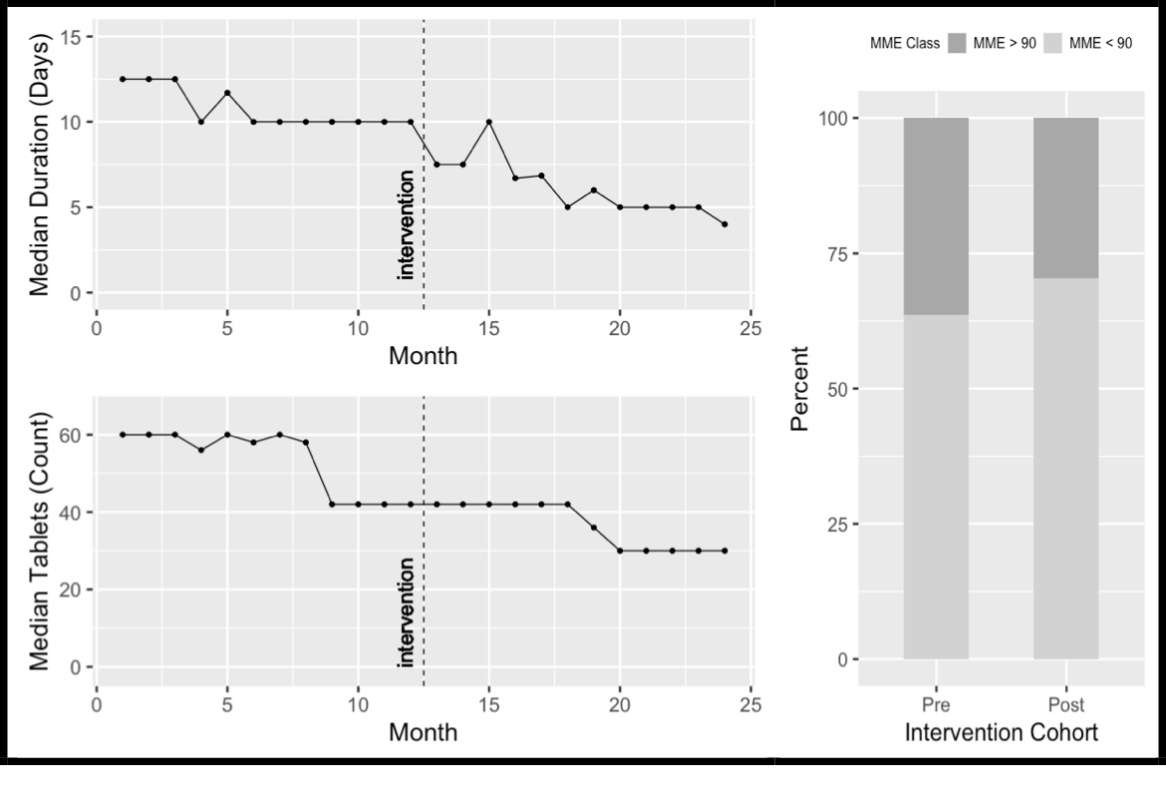
Monthly median number of tablets dispensed, median durations (days) of treatment and proportion of oxycodone prescriptions > 90 MME/day pre and post-intervention. MME: morphine milligram equivalents.

Given the majority of prescriptions were for oxycodone, Figure 3 demonstrates monthly numbers of tablets dispensed, median duration of treatment, and proportion of prescriptions greater than 90 MME/day for oxycodone.

**Table 2:** Quantity, duration of treatment, and proportion of MME^a^/day >90 for all opioids and each individual opioid before and after the intervention.

Opioid		Before the intervention	After the intervention	*P* value
**All opioids**				
	Quantity in tablets (IQR)	54 (40-120)	42 (18-90)	<.001
	Duration in days (IQR)	10.5 (5.0-30.0)	7.5 (3.0-30)	<.001
	>90 MME/day, n/N (%); 95% CI	10,704/38,976 (27.46); 27.02-27.91	8979/39,270 (22.86); 22.45-23.28	<.001
**Codeine**				
	Quantity in tablets (IQR)	42 (40-42)	20 (18-42)	<.001
	Duration in days (IQR)	5 (5-7.5)	2.5 (1.7-5.0)	<.001
	>90 MME/day, n/N (%); 95% CI	0	0	
**Hydrocodone**				
	Quantity in tablets (IQR)	42 (36-51)	45 (30-90)	.91
	Duration in days (IQR)	15 (11-22.5)	11.25 (8.75-15.63)	.53
	>90 MME/day, n/N (%); 95% CI	1/4 (25.00); 1.32-78.06	4/9 (44.44); 15.34-77.35	>.99
**Hydromorphone**				
	Quantity in tablets (IQR)	60 (42-120)	60 (20-120)	
	Duration in days (IQR)	7.5 (5-20)	7.5 (3.3-20)	
	>90 MME/day, n/N (%); 95% CI	783/1985 (39.45); 37.29-41.64	664/2010 (33.03); 30.99-35.15	<.001
**Meperidine**				
	Quantity in tablets (IQR)	144 (60-182)	100 (100-120)	.41
	Duration in days (IQR)	10.5 (5.0-15.0)	6 (6.0-10.5)	.93
	>90 MME/day, n/N (%); 95% CI	0	0	
**Morphine**				
	Quantity in tablets (IQR)	60 (56-90)	60 (60-90)	
	Duration in days (IQR)	30 (30-30)	30 (20-30)	
	>90 MME/day, n/N (%); 95% CI	1092/2456 (44.46); 42.49-46.46	1048/2335 (44.88); 42.85-46.93	.87
**Oxycodone**				
	Quantity in tablets (IQR)	56 (42-120)	42 (18-90)	<.001
	Duration in days (IQR)	10 (5.0-30.0)	6 (2.5-30)	<.001
	>90 MME/day, n/N (%); 95% CI	8722/23,962 (36.40); 35.79-37.01	7190/24,299 (29.59); 29.02-30.17	<.001
**Oxymorphone**				
	Quantity in tablets (IQR)	60 (56-90)	62 (60-90)	.18
	Duration in days (IQR)	30 (30-30)	30 (30-30)	
	>90 MME/day, n/N (%); 95% CI	85/143 (59.44); 50.90-67.47	58/96 (60.42); 49.89-70.10	>.99
**Tapentadol**				
	Quantity in tablets (IQR)	112 (60-166)	101 (60-120)	.29
	Duration in days (IQR)	30 (30-30)	30 (30-30)	
	>90 MME/day, n/N (%); 95% CI	21/82 (25.61); 16.89-36.65	15/90 (16.67); 9.93-26.32	.21
**Tramadol**				
	Quantity in tablets (IQR)	40 (28-97.5)	30 (12-90)	<.001
	Duration in days (IQR)	10 (7.5-20.0)	10 (5-20)	
	>90 MME/day, n/N (%); 95% CI	0	0	

^a^MME: morphine milligram equivalents.

## Discussion

### Principal Findings

In this quasi-experimental pre–post study our intervention resulted in a significant reduction (*P*<.001) in the duration of treatment, quantity of tablets dispensed, and proportion of prescriptions greater than 90 MME/day for all opioid prescriptions written for patients with acute pain without an increase in the rate of prescribing or MME/day per prescription, while maintaining clinician autonomy. Our postintervention median duration of treatment for all opioids was 7.5 days, which is slightly longer than the CDC’s recommendations of 7 days [16], but an improvement over our preintervention duration of 10 days. In addition, we improved compliance with CDC recommendations that opioid dosing should not exceed 90 MME/day [16].

Oxycodone accounted for more than 60% (48,261/78,246, 61.68%) of our institutions prescriptions and was the only opioid to demonstrate reductions in all metrics, while tramadol, codeine, and hydromorphone had reductions in at least one metric. Our results show an improved median duration of treatment for oxycodone of 6 days, which is within CDC’s recommendations for acute pain.

Modifications to prescription presets is a relatively simple and effective way to combat the opioid epidemic. Our results are similar to other studies of single departments. Delgado et al [23] studied 2 EDs prescribing oxycodone. They examined the difference in prescribing patterns before and after the implementation of a new EHR, where they previously had no prescribing presets and the new EHR was preset to 10 tablets per prescription demonstrating a decrease in the median number of tablets from 11.3 and 12.6 to 10 and 10.9, respectively, in the 2 departments [23].

Chiu et al [22] looked at opioid prescribing in the outpatient surgical setting. Their study included 3 hospitals examining changes in preset opioid prescription quantities from 30 to 12 tablets. The intervention reduced the median number of tablets prescribed from 30 to 20, with a decrease of 5.22 tablets and 34.41 MME per prescription, and no difference in refill rates [22].

Despite their expectation of decreased tablets dispensed, Zwank et al [26] showed an increase from 15.31 to 15.77 in the mean number of tablets dispensed for hydrocodone and oxycodone in the ED after they removed a 15-tablet preset and required manual entry of a dispense quantity [26]. This may suggest that presets are vital to reduce prescribing quantities; however, this is contradicted by Santistevan et al [24] who demonstrated that removal of a default of 20 tablets for hydrocodone and oxycodone reduced the median number of tablets prescribed from 20 to 15, although their preintervention presets were higher than Zwank et al’s.

Montoy et al [25] implemented a block-randomization study at 2 EDs. They examined 6 possible opioid tablet quantity presets: “status quo” 12 and 20, as well as “null,” 5, 10, and 15. They demonstrated that each tablet increase in preset yielded an increase of 0.19 tablets prescribed, and lower default quantities were associated with lower number of pills in 8 of 15 pairwise comparisons [25]. These results support our approach that modifications to prescription presets are an effective way to enact change in opioid prescribing.

Despite a number of publications demonstrating reductions in opioid prescribing through presets [22,23,25], to our knowledge this research is the first to demonstrate reduction in overall opioid prescribing and prescribing of multiple individual opioids for an entire hospital system. Our hospitals and ambulatory clinics are located in one of the most lethal counties in one of the most lethal states associated with the opioid epidemic [38-40]. The CDC estimates that the risk of chronic use of opioids is 13.5% after 8 days of treatment [15] and the World Health Organization estimates the annual rate for opioid-dependent individuals overdosing at 45% and death at 0.65% [41]. We reduced our median duration of treatment from 10.5 to 7.5 (6 days for oxycodone), thus potentially bringing more than half of our institutions’ opioid prescriptions below this high-risk threshold.

Examining our hospital’s nearly 3300 prescriptions per month and postintervention median reduction of 12 tablets per prescription, we can estimate that our hospital system has reduced nearly 39,600 opioid tablets from being prescribed per month, or 475,200 tablets per year. Extrapolate this to the over 168 million prescriptions written in the United States in 2018 [42], and our simple intervention could potentially reduce the number of tablets being prescribed each year by over 2 billion. Our intervention excluded prescriptions for chronic pain, thus these approximations are likely extreme, but even a small percentage of these estimates is a net large and clinically important reduction in opioid tablets. Significant reductions in the number of opioid tablets prescribed, durations of treatment, and doses greater than 90 MME/day can play an important role in curbing the epidemic, improving quality of care, and ultimately saving lives.

### Limitations

Our findings have a number of limitations. First, although a validated algorithm, we did not complete Tian et al’s [30] algorithmic approach to identifying patients with chronic pain with EHR data. We believe this is unlikely to significantly bias our results as this was the least sensitive element in the original study and exclusion of this step, if anything, biases our results away from our objective (ie, including more patients with chronic pain with presumably higher doses and longer prescription durations in our data set). In addition, this algorithm was designed for use with ICD-9 codes. We mapped ICD-9 to ICD-10 codes and performed a manual review, but errors may still have persisted. Second, with regard to our results, we did demonstrate that morphine and oxymorphone had slight reductions in proportion prescribed after the intervention; however, this was unlikely to have biased our results. We also should acknowledge that 9.16% (7167/78,246) of our study cohort were missing calculated durations and quantity of tablets dispensed and the percentage of these missing values decreased after the intervention, while 1.46% (1139/78,246) of prescriptions were missing MME/day and this increased after the intervention. We believe the majority of missing calculated durations are likely due to the missing dispense quantities, which are required for the computation of this metric. Missing MME/day is likely due to missing concentrations, doses, or conversion factors of some formulations. Some of these missing values may be due to prescriptions being refilled and bypassing some of the new requirements we implemented. We excluded missing data from our analysis, but it is possible this exclusion influenced our results.

For our MME/day threshold we chose to use the CDC guidelines for opioid prescribing as the CDC is a federal agency that upholds “the health of the people of the United States.” [43] It should be noted that individual states may have opioid prescribing guidelines that deviate from those presented by the CDC [44].

It should also be noted that our research was a quasi-experimental pre–post study, and we cannot infer causality with our intervention and the results observed. It is possible that other influential factors could have played a role in the reduction of opioid prescribing at out institution during this 2-year period, as the opioid epidemic has come to the forefront of modern medicine. However, we believe our results are encouraging, and other studies have demonstrated similar outcomes to suggest our intervention likely played at least a role in the reduction of prescribing patterns at our institution. Finally, our region has one of the highest opioid overdose and death rates in the nation, making our results of high value to curbing the epidemic; however, our results may not be generalizable to the rest of the country.

### Conclusions

In this quasi-experimental retrospective pre–post analysis, we demonstrated that modifications of the opioid prescribing presets in our hospital system’s EHR can improve prescribing practice patterns and reduce the number of pills dispensed, duration of treatment, and proportion of prescriptions greater than 90 MME/day. We have identified a simple and effective way to reduce opioid prescribing, and to our knowledge, we are the first to perform this kind of an intervention throughout an entire hospital system, and not just at the department level. Reduction in opioid prescribing may aid in curbing the opioid epidemic, thus improving quality of care and potentially saving lives.

## Appendix

Multimedia Appendix 1 Prior Opioid Settings.

## Abbreviations

CDC: Centers for Disease Control and Prevention
CPOE: computerized provider order entry
ED: emergency department
EHR: electronic health record
ICD-9: International Classification of Diseases, 9th Revision
MAT: medical assisted treatment
MME: morphine milligram equivalents

## References

[1] Srivastava AB, Gold MS (2018). Beyond Supply: How We Must Tackle the Opioid Epidemic. Mayo Clin Proc.

[2] US Department of Health and Human Services (2016). The Opioid Epidemic: By the Numbers.

[3] Rudd RA, Seth P, David F, Scholl L (2016). Increases in Drug and Opioid-Involved Overdose Deaths - United States, 2010-2015. MMWR Morb Mortal Wkly Rep.

[4] Rummans TA, Burton MC, Dawson NL (2018). How Good Intentions Contributed to Bad Outcomes: The Opioid Crisis. Mayo Clin Proc.

[5] American Pain Society Quality of Care Committee (1995). Quality improvement guidelines for the treatment of acute pain and cancer pain. JAMA.

[6] A consensus statement from the American Academy of Pain Medicine and the American Pain Society (1997). The use of opioids for the treatment of chronic pain. Clin J Pain.

[7] Van Zee A (2009). The promotion and marketing of oxycontin: commercial triumph, public health tragedy. Am J Public Health.

[8] Purdue Pharma (2007). Execs to pay $634.5 million fine in OxyContin case.

[9] Nora D, Volkow MD (2014). America's addiction to opioids: heroin and prescription drug abuse.

[10] (2017). Annual surveillance report of drug-related risks and outcomes.

[11] Jones CM (2013). Heroin use and heroin use risk behaviors among nonmedical users of prescription opioid pain relievers - United States, 2002-2004 and 2008-2010. Drug Alcohol Depend.

[12] Scholl L, Seth P, Kariisa M, Wilson N, Baldwin G (2018). Drug and Opioid-Involved Overdose Deaths — United States, 2013–2017. MMWR Morb. Mortal. Wkly. Rep.

[13] Bohnert ASB, Valenstein M, Bair MJ, Ganoczy D, McCarthy JF, Ilgen MA, Blow FC (2011). Association between opioid prescribing patterns and opioid overdose-related deaths. JAMA.

[14] Edlund MJ, Martin BC, Russo JE, DeVries A, Braden JB, Sullivan MD (2014). The role of opioid prescription in incident opioid abuse and dependence among individuals with chronic noncancer pain: the role of opioid prescription. Clin J Pain.

[15] Shah A, Hayes CJ, Martin BC (2017). Characteristics of Initial Prescription Episodes and Likelihood of Long-Term Opioid Use - United States, 2006-2015. MMWR Morb Mortal Wkly Rep.

[16] Dowell D, Haegerich TM, Chou R (2016). CDC Guideline for Prescribing Opioids for Chronic Pain--United States, 2016. JAMA.

[17] Dowell D, Haegerich TM, Chou R (2016). CDC Guideline for Prescribing Opioids for Chronic Pain — United States, 2016. MMWR Recomm. Rep.

[18] (2018). Percent of Hospitals, By Type, that Possess Certified Health IT.

[19] Parasrampuria S, Blanco M, Barker W (2017). Electronic Prescribing of Controlled Substances among Office-Based Physicians.

[20] Wen H, Hockenberry JM, Jeng PJ, Bao Y (2019). Prescription Drug Monitoring Program Mandates: Impact On Opioid Prescribing And Related Hospital Use. Health Aff (Millwood).

[21] (2018). What is computerized provider order entry?. https://www.healthit.gov/faq/what-computerized-provider-order-entry.

[22] Chiu AS, Jean RA, Hoag JR, Freedman-Weiss M, Healy JM, Pei KY (2018). Association of Lowering Default Pill Counts in Electronic Medical Record Systems With Postoperative Opioid Prescribing. JAMA Surg.

[23] Delgado MK, Shofer FS, Patel MS, Halpern S, Edwards C, Meisel ZF, Perrone J (2018). Association between Electronic Medical Record Implementation of Default Opioid Prescription Quantities and Prescribing Behavior in Two Emergency Departments. J Gen Intern Med.

[24] Santistevan JR, Sharp BR, Hamedani AG, Fruhan S, Lee AW, Patterson BW (2018). By Default: The Effect of Prepopulated Prescription Quantities on Opioid Prescribing in the Emergency Department. West J Emerg Med.

[25] Montoy JCC, Coralic Z, Herring AA, Clattenburg EJ, Raven MC (2020). Association of Default Electronic Medical Record Settings With Health Care Professional Patterns of Opioid Prescribing in Emergency Departments: A Randomized Quality Improvement Study. JAMA Intern Med.

[26] Zwank MD, Kennedy SM, Stuck LH, Gordon BD (2017). Removing default dispense quantity from opioid prescriptions in the electronic medical record. Am J Emerg Med.

[27] Slovis B, London K, Randolph F, Aini M, Mammen P, Martino C, Christopher T, Hollander J (2018). 336 The Effect of Implementing Electronic Health Record Default Prescribing Preferences on Opioid Prescriptions Written in the Emergency Department. Annals of Emergency Medicine.

[28] (2019). About Us: Thomas Jefferson University.

[29] (2019). Medication and Counseling Treatment.

[30] Tian TY, Zlateva I, Anderson DR (2013). Using electronic health records data to identify patients with chronic pain in a primary care setting. J Am Med Inform Assoc.

[31] World Health Organization (1978). International Classification of Diseases (9th Revision).

[32] World Health Organization (1992). The ICD-10 classification of mental and behavioural disorders: Clinical descriptions and diagnostic guidelines.

[33] (2009). Diagnosis Code Set General Equivalence Mappings: ICD-10-CM to ICD-9-CM and ICD-9-CM to ICD-10-CM 2009 Version: Documentation and User's Guide.

[34] Wang WLY, Yan J (2018). touch: Tools of Utilization and Cost in Healthcare.

[35] Slovis BH, Kairys J, Babula B, Girondo M, Martino C, Roke LM, Riggio J (2020). Discrepancies in Written Versus Calculated Durations in Opioid Prescriptions: Pre-Post Study. JMIR Med Inform.

[36] Drug Enforcement Administration‚ Department of Justice (2014). Schedule of controlled substances: placement of tramadol into schedule IV. Final rule. Fed Regist.

[37] Drug Enforcement Administration‚ Department of Justice (2014). Schedules of controlled substances: rescheduling of hydrocodone combination products from schedule III to schedule II. Final rule. Fed Regist.

[38] (2019). Drug Overdose Mortality by State.

[39] Estimated Pennsylvania Drug Overdose Deaths by County, 2017-2018.

[40] (2018). DEA Intelligence Report: Opioid Threat in Pennsylvania: Drug Enforcement Administration.

[41] World Health Organization (2018). Information sheet on opioid overdose.

[42] (2018). U.S. Opioid Prescribing Rate Maps.

[43] Centers for Disease Control and Prevention Mission Statement.

[44] Acute pain phase prescribing recommendations.

