# Supplementary material for: Reduction in Hospital System Opioid Prescribing for Acute Pain Through Default Prescription Preference Settings: Pre–Post Study
Source: J Med Internet Res. 2021 Apr 14;23(4):e24360. doi: 10.2196/24360 (PMC8082388; doi:10.2196/24360)
Supplement: Multimedia Appendix 1 [file jmir_v23i4e24360_app1.docx]

**Supplemental files**

Prior Opioid Settings.

| MEDICATION NAME | Duration | Dispense Quantity | Dispense Unit |  |
| --- | --- | --- | --- | --- |
| ACETAMINOPHEN 120 MG-CODEINE 12 MG/5 ML ORAL SOLUTION |  | 120 | mL |  |
| ACETAMINOPHEN 120 MG-CODEINE 12 MG/5 ML ORAL SUSPENSION |  | 120 | mL |  |
| ACETAMINOPHEN 300 MG-CODEINE 15 MG TABLET |  | 30 | tablet |  |
| ACETAMINOPHEN 300 MG-CODEINE 30 MG TABLET |  | 30 | tablet |  |
| ACETAMINOPHEN 300 MG-CODEINE 60 MG TABLET |  | 30 | tablet |  |
| BUPRENORPHINE HCL 0.3 MG/ML INJECTION SYRINGE |  | 1 | mL |  |
| BUTALBITAL 50 MG-ACETAMINOPHEN 325 MG-CAFFEINE 40 MG-CODEINE 30 MG CAP |  | 30 | capsule |  |
| BUTALBITAL COMPOUND-CODEINE 30 MG-50 MG-325 MG-40 MG CAPSULE |  | 30 | capsule |  |
| BUTORPHANOL TARTRATE 1 MG/ML INJECTION SOLUTION |  | 1 | mL |  |
| BUTORPHANOL TARTRATE 10 MG/ML NASAL SPRAY | 14 | 2.5 | mL |  |
| BUTORPHANOL TARTRATE 2 MG/ML INJECTION SOLUTION |  | 1 | mL |  |
| CODEINE SULFATE 15 MG TABLET | 10 | 30 | tablet |  |
| CODEINE SULFATE 30 MG TABLET | 10 | 30 | tablet |  |
| CODEINE SULFATE 60 MG TABLET | 10 | 30 | tablet |  |
| CODEINE-BUTALBITAL-ASA-CAFFEINE 30 MG-50 MG-325 MG-40 MG CAPSULE |  | 30 | capsule |  |
| FENTANYL 1,200 MCG LOZENGE ON A HANDLE | 7 | 30 | tablet |  |
| FENTANYL 1,600 MCG LOZENGE ON A HANDLE | 7 | 30 | tablet |  |
| FENTANYL 100 MCG BUCCAL TABLET, EFFERVESCENT | 7 | 28 | tablet |  |
| FENTANYL 100 MCG/HR TRANSDERMAL PATCH | 30 |  |  |  |
| FENTANYL 12 MCG/HR TRANSDERMAL PATCH | 30 |  | each |  |
| FENTANYL 200 MCG BUCCAL TABLET, EFFERVESCENT | 7 | 28 | tablet |  |
| FENTANYL 200 MCG LOZENGE ON A HANDLE | 7 | 30 | tablet |  |
| FENTANYL 25 MCG/HR TRANSDERMAL PATCH | 30 |  |  |  |
| FENTANYL 400 MCG BUCCAL TABLET, EFFERVESCENT | 7 | 28 | tablet |  |
| FENTANYL 400 MCG LOZENGE ON A HANDLE | 7 | 30 | tablet |  |
| FENTANYL 50 MCG/HR TRANSDERMAL PATCH | 30 |  |  |  |
| FENTANYL 600 MCG BUCCAL TABLET, EFFERVESCENT | 7 | 28 | tablet |  |
| FENTANYL 600 MCG LOZENGE ON A HANDLE | 7 | 30 | tablet |  |
| FENTANYL 75 MCG/HR TRANSDERMAL PATCH | 30 |  |  |  |
| FENTANYL 800 MCG BUCCAL TABLET, EFFERVESCENT | 7 | 28 | tablet |  |
| FENTANYL 800 MCG LOZENGE ON A HANDLE | 7 | 30 | tablet |  |
| HYDROCODONE 10 MG-ACETAMINOPHEN 325 MG TABLET |  | 30 | tablet |  |
| HYDROCODONE 10 MG-ACETAMINOPHEN 400 MG TABLET | 10 | 30 | tablet |  |
| HYDROCODONE 10 MG-ACETAMINOPHEN 500 MG TABLET | 10 | 30 | tablet |  |
| HYDROCODONE 10 MG-ACETAMINOPHEN 650 MG TABLET | 10 | 30 | tablet |  |
| HYDROCODONE 10 MG-ACETAMINOPHEN 750 MG TABLET | 10 | 30 | tablet |  |
| HYDROCODONE 2.5 MG-ACETAMINOPHEN 500 MG TABLET | 10 | 30 | tablet |  |
| HYDROCODONE 5 MG-ACETAMINOPHEN 325 MG TABLET |  | 30 | tablet |  |
| HYDROCODONE 5 MG-ACETAMINOPHEN 400 MG TABLET | 10 | 30 | tablet |  |
| HYDROCODONE 5 MG-ACETAMINOPHEN 500 MG CAPSULE | 10 | 30 | capsule |  |
| HYDROCODONE 5 MG-ACETAMINOPHEN 500 MG TABLET | 10 | 30 | tablet |  |
| HYDROCODONE 5 MG-IBUPROFEN 200 MG TABLET |  | 30 | tablet |  |
| HYDROCODONE 7.5 MG-ACETAMINOPHEN 325 MG TABLET |  | 30 | tablet |  |
| HYDROCODONE 7.5 MG-ACETAMINOPHEN 325 MG/15 ML ORAL SOLUTION |  | 120 | mL |  |
| HYDROCODONE 7.5 MG-ACETAMINOPHEN 400 MG TABLET | 10 | 30 | tablet |  |
| HYDROCODONE 7.5 MG-ACETAMINOPHEN 500 MG TABLET | 10 | 30 | tablet |  |
| HYDROCODONE 7.5 MG-ACETAMINOPHEN 500 MG/15 ML ORAL SOLUTION | 10 | 120 | mL |  |
| HYDROCODONE 7.5 MG-ACETAMINOPHEN 650 MG TABLET | 10 | 30 | tablet |  |
| HYDROCODONE 7.5 MG-ACETAMINOPHEN 750 MG TABLET | 10 | 30 | tablet |  |
| HYDROCODONE 7.5 MG-IBUPROFEN 200 MG TABLET |  | 30 | tablet |  |
| HYDROMORPHONE 2 MG TABLET | 10 | 30 | tablet |  |
| HYDROMORPHONE 4 MG TABLET | 10 | 30 | tablet |  |
| HYDROMORPHONE 50 MG/50 ML IN 0.9 % SODIUM CHLORIDE INJECTION |  | 25 | mL |  |
| HYDROMORPHONE 8 MG TABLET | 10 | 30 | tablet |  |
| IBUPROFEN-OXYCODONE 400 MG-5 MG TABLET | 10 | 30 | tablet |  |
| LEVORPHANOL TARTRATE 2 MG TABLET | 10 | 30 | tablet |  |
| MEPERIDINE 100 MG TABLET | 10 | 30 | tablet |  |
| MEPERIDINE 50 MG TABLET | 10 | 30 | tablet |  |
| METHADONE 10 MG TABLET | 10 | 30 | tablet |  |
| METHADONE 40 MG SOLUBLE TABLET | 10 | 30 | tablet |  |
| METHADONE 5 MG TABLET | 10 | 30 | tablet |  |
| MORPHINE (BULK) 100 % POWDER | 1 | 5 | g |  |
| MORPHINE (PF) 100 MG/100 ML (1 MG/ML) IN DEXTROSE 5 % IV |  | 250 | mL |  |
| MORPHINE 1 MG/ML INJECTION SYRINGE |  | 30 | mL |  |
| MORPHINE 10 MG HYPODERMIC TABLET | 10 | 30 | tablet |  |
| MORPHINE 15 MG IMMEDIATE RELEASE TABLET | 10 | 30 | tablet |  |
| MORPHINE 2 MG/ML INJECTION SOLUTION |  | 25 | mL |  |
| MORPHINE 30 MG IMMEDIATE RELEASE TABLET | 10 | 30 | tablet |  |
| MORPHINE BOLUS FROM BAG (COMFORT CARE - OPIOID NAIVE) IN D5W |  | 250 | mL |  |
| MORPHINE BOLUS FROM BAG (COMFORT CARE - OPIOID TOLERANT) 1 MG/ML IN D5W |  | 250 | mL |  |
| MORPHINE BOLUS FROM BAG (PAIN) 1 MG/ML IN D5W |  | 250 | mL |  |
| MORPHINE IN DEXTROSE 5 % 250 MG/250 ML INTRAVENOUS SOLUTION |  | 100 | mL |  |
| NALBUPHINE 10 MG/ML INJECTION SOLUTION |  | 1 | mL |  |
| NALBUPHINE 20 MG/ML INJECTION SOLUTION |  | 1 | mL |  |
| OXYCODONE 15 MG TABLET | 10 | 30 | tablet |  |
| OXYCODONE 30 MG TABLET | 10 | 30 | tablet |  |
| OXYCODONE 5 MG CAPSULE | 10 | 30 | capsule |  |
| OXYCODONE 5 MG TABLET | 10 | 30 | tablet |  |
| OXYCODONE-ACETAMINOPHEN 10 MG-300 MG TABLET | 10 | 30 | tablet |  |
| OXYCODONE-ACETAMINOPHEN 10 MG-325 MG TABLET | 10 | 30 | tablet |  |
| OXYCODONE-ACETAMINOPHEN 10 MG-400 MG TABLET | 10 | 30 | tablet |  |
| OXYCODONE-ACETAMINOPHEN 10 MG-500 MG TABLET | 10 | 30 | tablet |  |
| OXYCODONE-ACETAMINOPHEN 10 MG-650 MG TABLET | 10 | 30 | tablet |  |
| OXYCODONE-ACETAMINOPHEN 2.5 MG-300 MG TABLET | 10 | 30 | tablet |  |
| OXYCODONE-ACETAMINOPHEN 2.5 MG-325 MG TABLET | 10 | 30 | tablet |  |
| OXYCODONE-ACETAMINOPHEN 2.5 MG-400 MG TABLET | 10 | 30 | tablet |  |
| OXYCODONE-ACETAMINOPHEN 5 MG-300 MG TABLET | 10 | 30 | tablet |  |
| OXYCODONE-ACETAMINOPHEN 5 MG-325 MG TABLET | 10 | 30 | tablet |  |
| OXYCODONE-ACETAMINOPHEN 5 MG-400 MG TABLET | 10 | 30 | tablet |  |
| OXYCODONE-ACETAMINOPHEN 5 MG-500 MG CAPSULE | 10 | 30 | capsule |  |
| OXYCODONE-ACETAMINOPHEN 5 MG-500 MG TABLET | 10 | 30 | tablet |  |
| OXYCODONE-ACETAMINOPHEN 7.5 MG-300 MG TABLET | 10 | 30 | tablet |  |
| OXYCODONE-ACETAMINOPHEN 7.5 MG-325 MG TABLET | 10 | 30 | tablet |  |
| OXYCODONE-ACETAMINOPHEN 7.5 MG-400 MG TABLET | 10 | 30 | tablet |  |
| OXYCODONE-ACETAMINOPHEN 7.5 MG-500 MG TABLET | 10 | 30 | tablet |  |
| OXYMORPHONE 1.5 MG/ML INJECTION SOLUTION |  | 10 | mL |  |
| OXYMORPHONE 10 MG TABLET | 10 | 30 | tablet |  |
| OXYMORPHONE 5 MG RECTAL SUPPOSITORY | 10 | 6 | suppository |  |
| OXYMORPHONE 5 MG TABLET | 10 | 30 | tablet |  |
| PENTAZOCINE 50 MG-NALOXONE 0.5 MG TABLET | 10 | 30 | tablet |  |
| PENTAZOCINE LACTATE 30 MG/ML INJECTION SOLUTION |  | 10 | mL |  |
| PROPOXYPHENE 65 MG CAPSULE | 10 | 30 | capsule |  |
| PROPOXYPHENE COMPOUND-65 65 MG-389 MG-32.4 MG CAPSULE | 10 | 30 | capsule |  |
| PROPOXYPHENE N-ACETAMINOPHEN 100 MG-325 MG TABLET |  |  |  |  |
| PROPOXYPHENE N-ACETAMINOPHEN 100 MG-500 MG TABLET | 10 | 30 | tablet |  |
| PROPOXYPHENE N-ACETAMINOPHEN 100 MG-650 MG TABLET | 10 | 30 | tablet |  |
| PROPOXYPHENE N-ACETAMINOPHEN 50 MG-325 MG TABLET | 10 | 30 | tablet |  |
| PROPOXYPHENE NAPSYLATE 100 MG TABLET | 10 | 30 | tablet |  |
| PROPOXYPHENE-ACETAMINOPHEN 65 MG-650 MG TABLET | 10 | 30 | tablet |  |
| TRAMADOL 37.5 MG-ACETAMINOPHEN 325 MG TABLET | 5 | 30 | tablet |  |
| TRAMADOL 50 MG DISINTEGRATING TABLET |  |  |  |  |
| TRAMADOL 50 MG TABLET | 10 | 30 | tablet |  |
| TRAMADOL ER 100 MG TABLET,EXTENDED RELEASE 24 HR | 10 |  |  |  |
| TRAMADOL ER 200 MG TABLET,EXTENDED RELEASE 24 HR | 10 |  |  |  |
| TRAMADOL ER 300 MG TABLET,EXTENDED RELEASE 24 HR | 10 |  |  |  |
|  |  |  |  |  |
